# Molecular Cloning and Expression Profile of Class *E* Genes Related to Sepal Development in *Nelumbo nucifera*

**DOI:** 10.3390/plants10081629

**Published:** 2021-08-09

**Authors:** Zhuoxing Liu, Dasheng Zhang, Weiwei Zhang, Lei Xiong, Qingqing Liu, Fengluan Liu, Hanchun Li, Xiangjie An, Lijie Cui, Daike Tian

**Affiliations:** 1 Shanghai Key Laboratory of Plant Functional Genomics and Resources, Shanghai Chenshan Plant Science Research Center of Chinese Academy of Sciences, Shanghai Chenshan Botanical Garden, Shanghai 201602, China; 1000459190@smail.shnu.edu.cn (Z.L.); dszhang@cemps.ac.cn (D.Z.); 1000479715@smail.shnu.edu.cn (L.X.); nicecocoliu@sina.com (Q.L.); lihanchun1022@126.com (H.L.); anxiangjie@cemps.ac.cn (X.A.); 2 Development Center of Plant Germplam Resources, College of Life Science, Shanghai Normal University, Shanghai 200234, China; 19liu19@163.com; 3 Department of Plant Science and Technology, Shanghai Vocational College of Agriculture and Forestry, Shanghai 201699, China; zhangww@shafc.edu.cn

**Keywords:** MADS-box, *SEPALLATA*, *AGL9*, *MADS6-like*, sepal, petal, *Nelumbo*

## Abstract

The lotus (*Nelumbo* Adans.) is an important aquatic plant with ornamental, medicinal and edible values and cultural connotations. It has single-, semi-double-, double- and thousand-petalled types of flower shape and is an ideal material for developmental research of flower doubling. The lotus is a basal eudicot species without a morphological difference between the sepals and petals and occupies a critical phylogenetic position in flowering plants. In order to investigate the genetic relationship between the sepals and petals in the lotus, the class *E* genes which affect sepal formation were focused on and analyzed. Here, *SEPALLATA 1*(*NnSEP1*) and its homologous genes *AGAMOUS-LIKE MADS-BOXAGL9* (*NnAGL9*) and *MADS-BOX TRANSCRIPTION FACTOR 6-like* (*NnMADS6-like*) of the class *E* gene family were isolated from the flower buds of the Asian lotus (*Nelumbo nucifera* Gaertn.). The protein structure, subcellular localization and expression patterns of these three genes were investigated. All three genes were verified to locate in the nucleus and had typical MADS-box characteristics. *NnSEP1* and *NnMADS6-like* were specifically expressed in the sepals, while *NnAGL9* was highly expressed in the petals, suggesting that different developmental mechanisms exist in the formation of the sepals and petals in the lotus. The significant functional differences between NnSEP1, NnMADS6-like and NnAGL9 were also confirmed by a yeast two-hybrid assay. These results expand our knowledge on the class *E* gene family in sepal formation and will benefit fundamental research on the development of floral organs in *Nelumbo.*

## 1. Introduction

Plant floral identity organs are controlled by a series of homeomorphic genes and almost allomorphic genes related to flower organ development that belong to the family of MADS-box genes [1]. The MADS-box gene family has become an essential part of the formation of plant floral organs. The MADS-box gene family is a crucial transcription factor with a highly conserved MADS domain that widely presents in various organisms [2,3,4]. The MADS-box gene family can be divided into two groups: type I and type II [5]. The type II genes comprise the MIKC-type genes of plants and the MEF2-like genes of animals and fungi. Moreover, the MIKC-type proteins own an extremely conserved MADS domain and three moderately conserved domains (I, K and C) [6]. The various members of the MADS-box gene family have been known to participate predominantly in developmental processes, such as plant vegetative reproduction [7,8], flower organ formation [9,10,11], male and female gametophyte development [12] and fruit development [13].

An ABC model of flower development was proposed by a previous report [14]. The ABC molecular regulation model indicates that the four whorl organs from the outside to the inside are controlled by three types of gene. For example, the *A* gene controls the sepals alone, *A* + *B* control the petals, *B* + *C* control the stamens and *C* controls the pistils alone. Simultaneously, the *A* and *C* genes repress each other [14]. Then, the ABCDE model and the quartet model of flower development were proposed and widely recognized by researchers [15,16,17]. The ABCDE model proposes that floral organs can be subdivided into five different classes of sepals, petals, stamens, pistils and ovules. The formation of flower organs depends on a protein tetramer encoded by five types of genes. The quartet model could accurately describe the interaction of five types of transcription factors. It also could explain the mechanism of flower phenotypes because of the loss of transcription factor genes [17].

Class *E* genes are central to the sepals and to regulating the formation of floral organs in each round. The *SEPALLATA* (*SEP*) gene belongs to class *E* and is a separate subfamily of the MADS-box gene family [18,19]. Previous studies showed that the triple mutants *SEP1/2/3* in *Arabidopsis* caused all flower organs to turn into sepals. Additionally, the *SEP* gene could shorten the vegetative growth period and promote the early flowering of plants [11,18,20,21,22]. Besides the genes in the ABCDE model, other MADS-box genes are also responsible for sepal development. The *AGL6* gene subfamily in plants is ancient and widespread [23]. Phylogenetic analysis shows that the *AGL6* homologous gene belongs to the super branch of *AP1/AGL9* (including *AGL6*, *SEP* and *SQUA*) [24]. The two branches of *AGL6* and *SEP* represent sister relationships to each other, and the domains at the C-terminal are extremely similar [25]. It has been speculated that the *AGL6* homologous gene and the *SEP* gene are similar in function [26].

**Figure 1:**
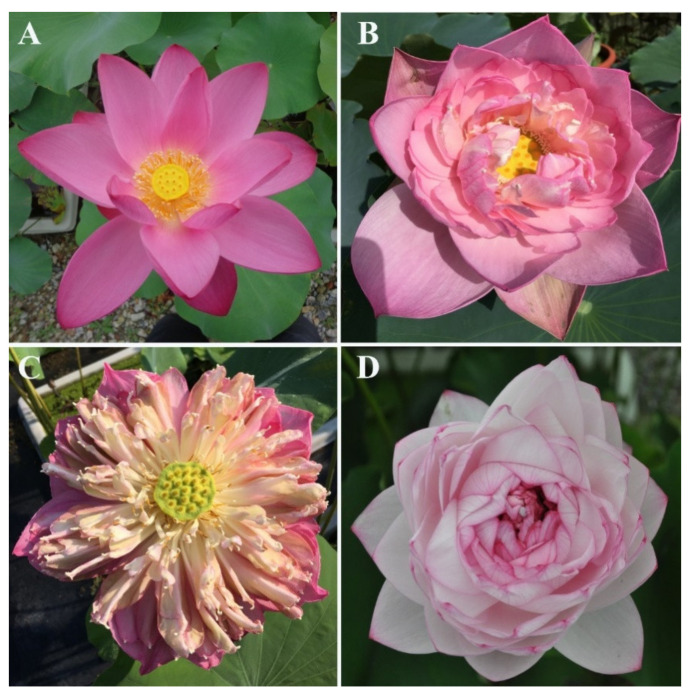
The floral forms of *Nelumbo nucifera*. (**A**) Single form; (**B**) semi-double form; (**C**) double form; (**D**) thousand-petalled form.

The lotus (*Nelumbo* Adans.) is a perennial aquatic plant and one of the top ten most famous flowers in China. It is an important crop with ornamental, medicinal and edible uses and cultural connotations. In addition, it is one of the earlier plants in the origin of angiosperms and is considered as a ‘living fossil’. The lotus is divided into two species according to its geographical location and morphological differences: the American lotus (*N. lutea* Willd.), and the Asian lotus (*N. nucifera* Gaertn.). The American lotus has a single flower only, while the Asian lotus has a variety of floral shapes, including single-, semi-double-, double- and thousand-petalled types (Figure 1). No reproductive isolation occurs between the two species and among most cultivars of the lotus; therefore, it is a good material for studying floral development. In *Nelumbo*, the phenotype of the outermost sepals (usually four) is similar to the inner petals, meaning breeders usually use the concept of ‘tepal’ instead of ‘sepal’ for petals in the lotus. In order to investigate the genetic relationship between sepals and petals in the lotus, the class *E* genes, which affect sepal formation, were focused on and analyzed. In this study, a single-petalled type of wild Asian lotus was used as the experimental material. Using the RT-PCR method, the *SEP* gene and its homologous genes relating to sepal formation were cloned, and a series of bioinformatics and function analyses was performed.

## 2. Materials and Methods

### 2.1. Experimental Materials

The wild type of the Asian lotus (Figure S1) was used as the experimental material. It was collected from Weishan Lake in Shandong Province, China, and cultivated in the International Nelumbo Collection, which is located at Shanghai Chenshan Botanical Garden. The root, pedicel, leaf, petal, stamen, pistil, receptacle and rhizome of the Asian lotus at the flower maturation stage were sampled for analysis of genes’ expression patterns. Following sample collection, all the samples were immediately treated by liquid nitrogen freezing and stored at −80 °C for subsequent uses.

### 2.2. Total RNA Extraction and cDNA Synthesis

The RNA of the root, pedicel, leaf, petal, stamen, pistil, receptacle, rhizome and flower was extracted according to the instructions of the RNAprep Pure Plant Kit (TIANGEN, DP441, Beijing, China). The quality and purity of RNA samples were determined using the Thermo ND2000c (Thermo Fisher Scientific, American). The reverse transcription reaction was performed according to the instructions of the High Efficiency Reverse Transcription Kit (TOYOBO, FSK-100, Osaka, Japan).

### 2.3. Gene Clone and Sequence Identification

From the NCBI (https://www.ncbi.nlm.nih.gov/, accessed on 9 January 2021), only one *SEP* gene (XM_010259656) was predicted to belong to the class *E* gene family. The total RNA of the Asian lotus flower bud (<2 cm) was used as a template for high-fidelity amplification. After the reverse transcription with the primer AP, *NnSEP1*-full-F and AUAP were designed to amplify the full length of the *SEP* gene with KOD-Plus public high-fidelity kit (KOD-201, Japan) (the primers are listed in Table 1). The PCR products were sequenced by Qingke Biotechnology Company (Shanghai, China).

### 2.4. Sequence Analysis

Homologous sequence analysis was performed online using Blast provided by NCBI, and the open reading frames of cloned genes were searched online using ORF Finder. Sequence analysis and amino acid translation were performed using DNAMAN 6.0 software. InterPro (http://www.ebi.ac.uk/interpro/search/sequence, accessed on 1 February 2021) was used to predict the protein domain. PtotParam provided by ExPASY (https://expasy.org/proteomics, accessed on 5 February 2021) was used to analyze the physicochemical properties of the protein online. ProtScale was used to analyze the hydrophobicity of the protein. NetPhos 2.0 Server (http://www.cbs.dtu.dk/services/NetPhos-2.0/, accessed on 6 February 2021) was used to predict the phosphorylation site. GOR4 was used to predict the secondary structure of the protein. SWISS-MODEL was used to predict the tertiary structure of the protein. PSORT (https://www.genscript.com/tools/psort, accessed on 18 February 2021) provided by GenScript was used to perform subcellular localization prediction online. TMHMM (http://www.cbs.dtu.dk/services/TMHMM/, accessed on 5 February 2021) was used to perform transmembrane domain analysis. DNAMAN 6.0 software was used for multiple sequence alignment, and MAGE 5.0 software was used to construct the phylogenetic tree.

### 2.5. Subcellular Localization

The full length of *NnSEP1*, *NnAGL9* and *NnMADS6-like* genes without an amino acid stop code was amplified by PCR (primers are listed in Table S1). Then, the fragments were digested with *Bam*H I, and *Xba* I was ligated with the pCAMBIA 1300-*GFP* vector with the same digestion to construct the fusion vectors pCAMBIA1300-*NnSEP1-GFP*, pCAMBIA1300-*NnAGL9-GFP* and pCAMBIA1300-*NnMADS6L-GFP*. The fusion plasmids were transformed into *Agrobacterium GV3101* competent cells, and the positive clones were selected for transformation into *Nicotiana benthamiana* for the transient expression test. *Nicotiana benthamiana* was grown in a greenhouse under the conditions of 25 °C, 70% relative humidity and long day (16 h light and 8 h dark) for subcellular localization experiments. The pellets were resuspended in 200 mL MS containing 200 μM acetosyringone and 0.39 g MES at 28 °C and were incubated in a shaker at 140 rpm for 2–3 h until OD_600_ reached 0.6. Two *Agrobacterium GV3101* strains carrying the recombinant expression plasmid and control were injected into tobacco leaves. The treated *N. benthamiana* was cultured in the dark for 24 h and then in the light for 24 h at 25 °C. The dorsal epidermis of the leaf blade was collected, and the GFP fluorescence signal was detected by a laser confocal microscope (Olympus, FV10i), as described in a previous report [27].

### 2.6. Gene Expression Analysis

The gene expression in the root, stem, leaf, petal, stamen, pistil, receptacle, rhizome and flower buds was detected using the real-time quantitative RT-PCR method, using *NnACTIN* as the internal reference gene [28]. The RT-PCR primers are listed in Table 1. The quantitative reactions were performed based on the SYB Green design manual (TAKARA, DRR820A). The quantitative reaction was performed on an ABI Step ONE quantitative PCR instrument, using the (2^−ΔΔCT^) method to calculate the relative expression level. Each experiment was replicated three times (3 biological and 3 technical repeats).

### 2.7. Yeast Two-Hybrid (Y2H) Assay

A yeast two-hybrid assay of the GAL4 system was used, as described in a previous report [29]. The full-length coding sequences of *NnSEP1*, *NnAGL9* and *MADS6-like* were cloned and constructed into the pGBKT7 and pGADT7 vectors (the primers are listed in Table 1). The bait and prey fusion plasmid were co-transformed into strain *AH109* using the lithium acetate method. The isolated colonies were incubated on SD selection medium (SD/−Trp/−Leu and SD/−Trp/−Leu/−His/−Ade/+X-α-gal) plates in triplicates for interaction analysis.

## 3. Results

### 3.1. Cloning of the Full-Length cDNA of the SEP Gene

Only *SEP1* was predicted to belong to the class *E* gene family in the lotus genome database (https://www.ncbi.nlm.nih.gov/nuccore/XM_010259656.2, accessed on 9 January 2021). According to the sequence predicted by the database, the specific primers *NnSEP1*-full-F and AUAP were designed. After the reverse transcription with the primer AP, the designed *NnSEP1*-full-F and AUAP were used to amplify the full length of the *SEP1* gene (the primers are list in Table 1).

**Figure 2:**
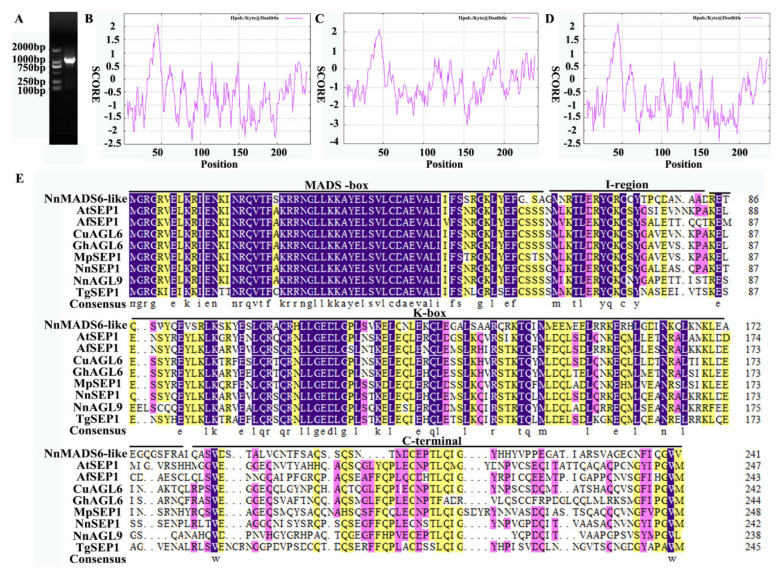
Cloning of three *NnSEP* genes and predictive analysis of their protein domains. (**A**) Cloning of the full-length cDNA of three *NnSEP* genes. (**B**) The prediction analysis of ProtScale protein hydrophobicity of NnSEP1. (**C**) The prediction analysis of ProtScale protein hydrophobicity of NnMADS6-like. (**D**) The prediction analysis of ProtScale protein hydrophobicity of NnAGL9. (**E**) Sequence alignment of SEP protein in *Nelumbo nucifera* and other species. The protein sequences of NnSEP1, NnMADS6-like and NnAGL9 aligned were retrieved from NCBI. Different species and matching GenBank accession numbers are shown below. AtSEP1 from *Arabidopsis thaliana* (AAU82007.1), AfSEP1 from *Aristolochia fimbriata* (ALV83431.1), CuAGL6 from *Citrus unshiu* (GAY42048.1), GhAGL6 from *Gossypium hirsutum* (XP_040972459.1), MpSEP1 from *Medicago polyceratia* (AFU81295.1), TgSEP1 from *Tulipa gesneriana* (AQR58150.1).

A single band of about 900 bp was obtained by the PCR method (Figure 2A), which was close to the size predicted in the lotus genome. After the band was recovered and cloned into the blunt vector, we sequenced it and found that this band was a mixture of several gene fragments, the *SEP1* gene and two new genes (*NnAGL9* and *NnMADS6-like*) with high homology to the *SEP1* gene.

The open reading frame (ORF) of the *NnSEP1* gene was 732 bp in length and encoded 243 amino acids. The BLAST analysis of the homologous sequence showed that the gene had homology with the *SEP1* gene of other plants. The ORF of the *NnAGL9* gene was 720 bp in length and encoded 239 amino acids. The BLAST analysis of the homologous sequence showed that the gene has homology with the *SEP3* gene in other plants. The ORF of the *NnMADS6-like* gene was 729 bp in length and encoded 242 amino acids. The BLAST analysis of the homologous sequence revealed that the *NnMADS6-like* gene had homology with other plant *AGL6* genes.

DNAMAN software was used to compare the amino acid sequences of NnSEP1, NnAGL9 and NnMADS6-like (Figure 2E). All three proteins had the characteristics typical of MADS-box genes. The MADS-box domain at the 5′ end was highly conserved, and the middle K-box domain was conserved, while the 3′C-terminus was quite different.

### 3.2. Analysis of Physicochemical Properties of Proteins

The molecular weights of the NnSEP1, NnAGL9 and NnMADS6-like proteins were all about 27 kDa, and their theoretical isoelectric points were 9.15, 8.76 and 9.12, respectively. The total average values of the three proteins’ hydrophilicity by ProtParam analysis were −0.653, −0.703 and −0.696, respectively. This indicates that they all remained with hydrophilic protein characters. The result of ProtScale software analysis was the same as that of ProtParam analysis. The prediction analysis of ProtScale protein hydrophobicity was divided according to the score (Figure 2B–D). The NnSEP1, NnAGL9 and NnMADS6-like proteins all remained with hydrophilic proteins. The maximum score of the three proteins was 2.122, locating at the 45th amino acid. The minimum values were −2.389, −2.422 and −3.033, locating at amino acids 89, 174 and 154, respectively. NetPhos 2.0 Server showed that there were 11 serine phosphorylation sites for NnSEP1, 1 threonine and 7 serine phosphorylation sites for NnAGL9 and 1 tyrosine and 9 serine phosphorylation sites for NnMADS6-like.

### 3.3. Secondary and Tertiary Structures of Proteins

**Figure 3:**
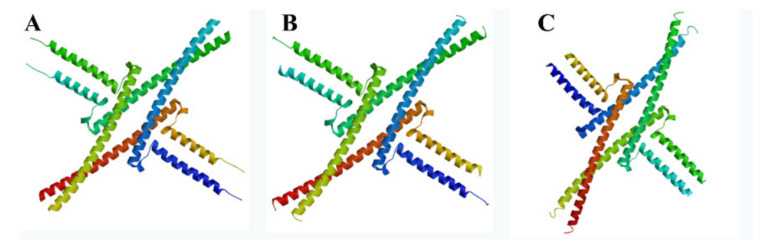
The 3D structural model of proteins. (**A**) NnSEP1; (**B**) NnAGL9; (**C**) NnMADS6-like.

Analysis by GOR4 software showed that the NnSEP1, NnAGL9 and NnMADS6-like proteins were composed of three typical secondary structures of α-helix, random coils and extended chains. Simultaneously, the α-helix structure accounts for more than 50%. The SWISS-MODEL platform was used to predict the tertiary structure of the three proteins, NnSEP1, NnAGL9 and NnMADS-like. The three proteins were mainly composed of an α-helix structure, which was consistent with the secondary structure prediction results (Figure 3). The structures of the NnSEP1, NnAGL9 and NnAGL6 proteins in the lotus were very similar.

### 3.4. Phylogenetic Analysis of NnSEP1, NnAGL9 and NnMADS6-Like Proteins

In order to determine the classification of the NnSEP1, NnAGL9 and NnMADS6-like proteins, the amino acid sequences of NnSEP1, NnMADS6-like and NnAGL9 were used as a template to query the protein database from the NCBI (http://www.ncbi.nlm.nih.gov/blast/Blast.cgi, accessed on 9 January 2021). Through sequence comparison, 32 protein sequences were selected with more than 68% identity to NnSEP1, NnMADS6-like and NnAGL9. Clustal W was used for aligning protein sequences. The phylogenetic relationship was finally analyzed using MEGA 5.0 software [30,31,32].

**Figure 4:**
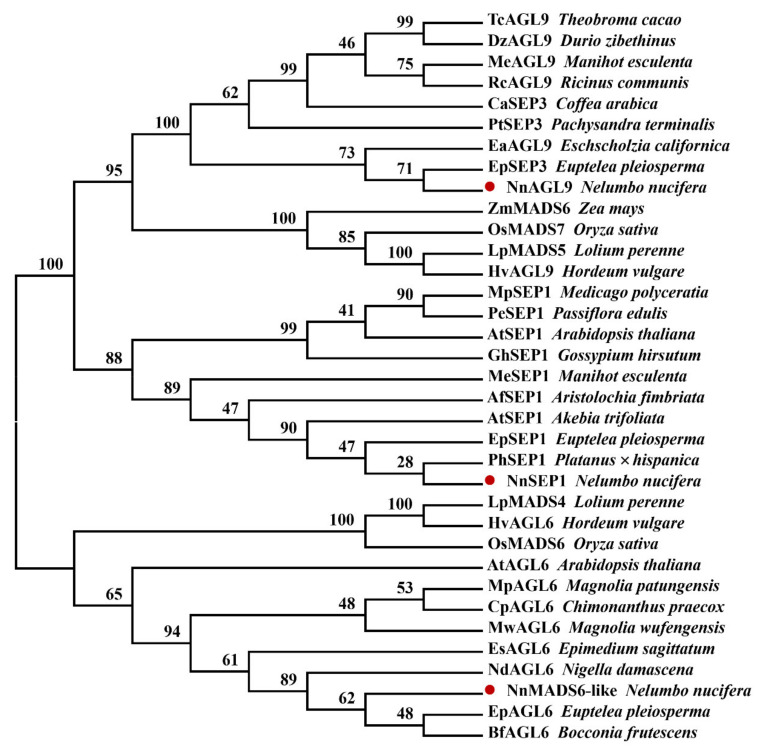
Phylogenetic analysis of NnSEP1, NnAGL9 and NnMADS6-like and their homologous sequences from various plant species.

The constructed phylogenetic trees were divided into two clans: NnMADS6-like and NnSEP1/NnAGL9 (Figure 4). NnSEP1′s closest genetic relationship is with SEP1 of *Platanus* × *hispanica* and *Euptelea pleiosperma*. NnAGL9 belongs to the SEP3 class, which was divided into two branches. One branch was monocotyledonous plants containing *Lolium perenne*, *Oryza sativa*, *Zea mays* and *Hordeum vulgare*. The other was dicotyledon. NnAGL9 was first clustered together with the SEP3 protein of *Euptelea pleiosperma* and then took part in a big branch with other dicotyledon SEP3 classes. Similarly, NnMADS6-like was also the first to gather together with the AGL6 protein of *Euptelea pleiosperma* and *Bocconia frutescens* and then joined a large branch with other dicotyledons.

The phylogenetic tree was constructed using the neighbor joining method. Boot strap percentages are shown at dendrogram branch points. The different species and matching GenBank accession numbers are as follows: TcAGL9 from *Theobroma cacao* (XP_007043947.1), DzAGL9 from *Durio zibethinus* (XP_022724906.1), MeAGL9 from *Manihot esculenta* (XP_021631286.1), RcAGL9 from *Ricinus communis* (XP_015572264.1), CaSEP3 from *Coffea arabica* (AHW58034.1), PtSEP3 from *Pachysandra terminalis* (ADC79703.1), EaAGL9 from *Eschscholzia californica* (AAX15918.1), EpSEP3 from *Euptelea pleiosperma* (ADC79706.1), ZmMADS6 from *Zea mays* (NP_001105153.1), OsMADS7 from *Oryza sativa* (P0C5B0.2), LpMADS5 from *Lolium perenne* (AAO45877.1), HvAGL9 from *Hordeum vulgare* (AAS48129.1), MpSEP1 from *Medicago polyceratia* (AFU81295.1), PeSEP1 from *Passiflora edulis* (AET98846.1), AtSEP1 from *Arabidopsis thaliana* (AAU82007.1), GhSEP1 from *Gossypium hirsutum* (AEL33631.1), MeSEP1 from *Manihot esculenta* (XP_021592871.1), AfSEP1 from *Aristolochia fimbriata* (ALV83431.1), AtSEP1 from *Akebia trifoliata* (ADC79694.1), EpSEP1 from *Euptelea pleiosperma* (ADC79707.1), PhSEP1 from *Platanus* × *hispanica* (ADR83588.1), LpMADS4 from *Lolium perenne* (AAO45876.1), HvAGL6 from *Hordeum vulgare* (AAS48128.1), OsMADS6 from *Oryza sativa* (Q6EU39.1), AtAGL6 from *Arabidopsis thaliana* (NP_182089.1), MpAGL6 from *Magnolia patungensis* (ATB53135.1), CpAGL6 from *Chimonanthus praecox* (ACN88212.1), MwAGL6 from *Magnolia wufengensis* (AOZ15901.1), EsAGL6 from *Epimedium sagittatum* (AEX58638.1), NdAGL6 from *Nigella damascena* (ALM95509.1), EpSEP1 from *Euptelea pleiosperma* (ADC79707.1), BfAGL6 from *Bocconia frutescens* (AOC50668.1).

### 3.5. Subcellular Localization of NnSEP1, NnAGL9 and NnMADS6-Like Proteins

**Figure 5:**
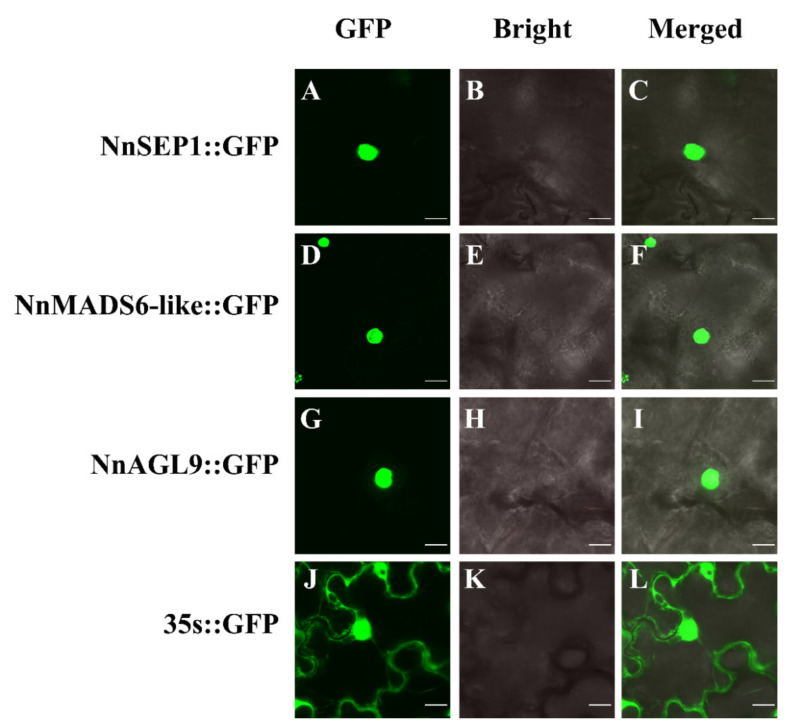
Subcellular localization of NnSEP1, NnMADS6-like and NnAGL9 in *N. benthamiana*. (**A**) 35S-NnSEP1-GFP was especially expressed in the nucleus of tobacco leaf cells. (**B**) The bright field of 35S-NnSEP1-GFP in tobacco leaf. (**C**) The merged field of GFP and the bright field of 35S-NnSEP1-GFP. (**D**) 35S-NnMADS6-like-GFP was especially expressed in the nucleus. (**E**) The bright field of 35S- NnMADS6-like-GFP in tobacco leaf. (**F**) The merged field of GFP and the bright field of 35S- NnMADS6-like-GFP. (**G**) 35S-NnAGL9-GFP was especially expressed in the nucleus. (**H**) The bright field of 35S-NnAGL9-GFP in tobacco leaf. (**I**) The merged field of GFP and the bright field of 35S-NnAGL9-GFP. (**J**) 35S-GFP was especially expressed in the nucleus and plasma membranes. (**K**) The bright field of 35S-GFP in tobacco leaf. (**L**) The merged field of GFP and the bright field of 35S- GFP. Bar = 50 μm.

Prediction analysis of the subcellular localization with PSORT showed that NnSEP1, NnAGL9 and NnMADS6-like were mainly distributed in the nucleus. The TMHMM transmembrane domain speculated that neither NnSEP1, NnMADS6-like nor NnAGL9 had a transmembrane signal peptide. Such localization patterns are consistent with the existence of the MADS-box gene family as the transcription factors. To further confirm this, NnSEP1, NnMADS6-like and NnAGL9 were fused with the green fluorescent protein (GFP) and transformed into tobacco leaves for localization analysis. After observation under a laser confocal microscope, NnSEP1, NnAGL9 and NnMADS6-like were determined to be located in the nucleus (Figure 5).

### 3.6. Expression Pattern Analysis of NnSEP1, NnAGL9 and NnMADS6-like Genes

In order to analyze the gene expression in the different plant tissues, we collected different parts of the single-flowered Asian lotus at the bud stage, including the root, pedicel, leaf, receptacle, rhizome and flower. All three genes were specifically expressed in the flower but hardly expressed in other organs (Figure S2). To further clarify the expression patterns of the three genes in floral tissues, we collected flower buds at four different growth stages (Figure S3) and divided them into sepals, petals, stamens and pistils for detection.

**Figure 6:**
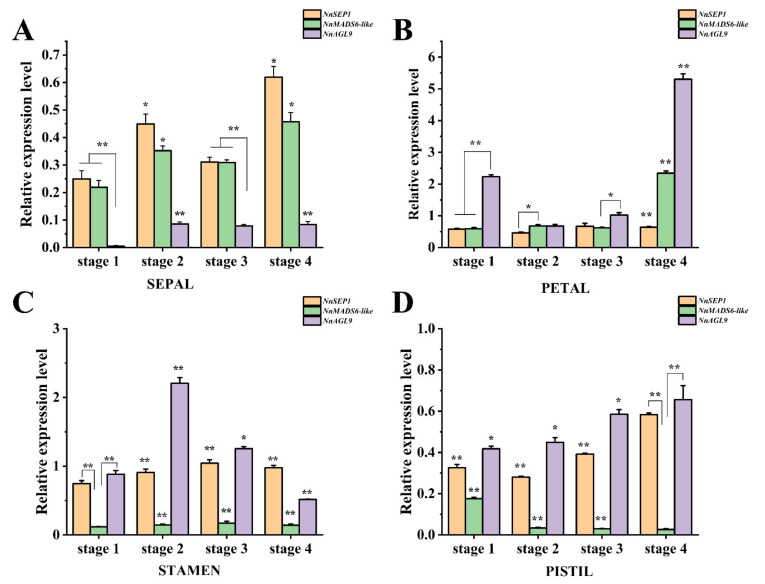
The expression pattern of three genes in different tissues at different floral development stages of *N. nucifera.* (**A**) Expression pattern of three genes in sepals. (**B**) Expression pattern of three genes in petals. (**C**) Expression pattern of three genes in stamens. (**D**) Expression pattern of three genes in pistils. The genes expression was detected using the RT-PCR method and *NnACTIN* as the internal reference gene. All data are means of three replicates, with error bars indicating standard deviations. The values are means ± SD of triplicate analysis (* indicating *p* < 0.05, ** indicating *p* < 0.001).

In the sepals, the expression patterns of the three genes were similar, but the expression of *NnAGL9* was the lowest of the three genes (Figure 6A), which suggests that *SEP1* and *MADS6-like* play an important role in sepal formation. In the petals, the expression level of *NnMADS6-like* and *NnAGL9* reached the highest level at the mature stage. However, the expression of *NnSEP1* did not change markedly in each period (Figure 6B). Expression patterns were similar for *NnSEP1* and *NnAGL9* in the stamens and pistils, while *NnMADS6-like* was barely expressed in both tissues. In the stamens, the expression of *NnMADS6-like* was low, and the expression tendency of *NnSEP1* and *NnAGL9* was different in other tissues. The expression level of *NnAGL9* reached the highest at stage II and the lowest at stage IV (Figure 6C). Although the expression of the genes in the pistils slightly fluctuated, their expression was not as high as in the stamens (Figure 6D).

### 3.7. Protein Interaction Between NnSEP1, NnMADS6-Like and NnAGL9

**Figure 7:**
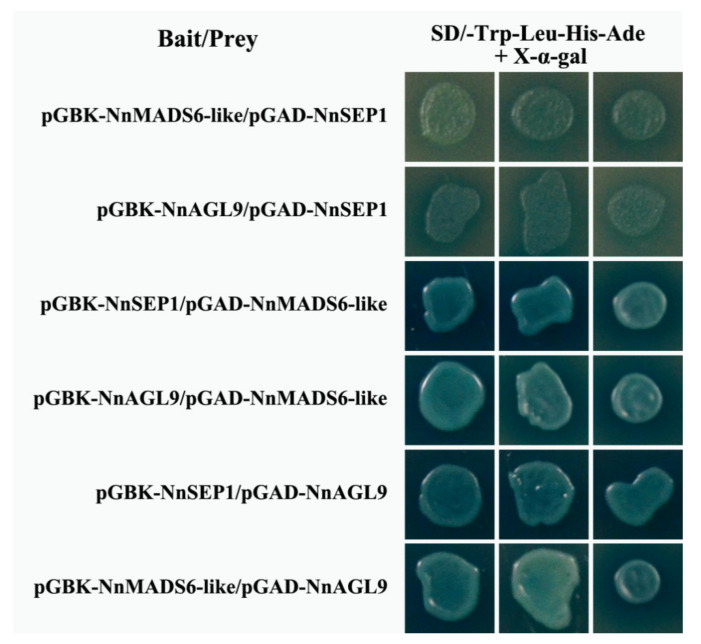
Protein interactions between NnSEP1, NnMADS6-like and NnAGL9 in vitro. Interactions were determined in yeast on selective medium SD/-Trp-Leu-His-Ade/X-α-Gal, and the experiments were repeated three times with the same results. AD and BK clones containing empty vectors were used as the negative controls, shown in Figure S4.

Class *E* genes play a fundamental role in floral organogenesis by binding different types of proteins. To identify the protein–protein interactions among NnSEP1, NnMADS6-like and NnAGL9, an in vitro yeast two-hybrid (Y2H) assay was introduced to this study. The result shows NnMADS6-like interacted with NnAGL9 in yeast, no matter who was the bait. However, NnSEP1 can bind to NnMADS6-like and NnAGL9 when using NnSEP1 as the bait. On the contrary, NnSEP1 could not form a polymer when it was used as the prey (Figure 7). This infers that NnMADS6-like and NnAGL9 can form a complex interaction during the development of floral organs, while the function of NnSEP1 was different from NnMADS6-like and NnAGL9. However, more experimental evidence is needed to understand its biological function.

## 4. Discussion

Floral organs are pivotal for flowering plants and controlled by intricate genetic networks. The MADS-box genes have been recruited as the primary elements of the genetic networks that control flower organ formation during plant evolution. Although several genes involved in lotus flower formation have been predicted, none of them, however, have been cloned and characterized with any function [33,34]. In this work, in order to understand the genetic relationship between the sepals and petals of the lotus, we cloned an *E* class gene, *NnSEP1*. Unexpectedly, and fortunately, we also obtained two homologous genes *NnAGL9* and *NnMADS6-like* from *N. nucifera*. Sequence homology analysis showed that *NnSEP1*, *NnAGL9* and *NnMADS6-like* belonged to the MADS-box gene family. Their 5′-end sequences were very similar, but the 3′-end sequences were different. The expression pattern showed a significant difference in function between these two genes and *SEP1*. The localization prediction and subcellular localizations of NnSEP1, NnAGL9 and NnMADS6-like showed that all three proteins were located in the nucleus. Previous studies showed that there were three *SEP* genes (*SEP1*, *SEP2* and *SEP3*) in *Arabidopsis* which affected the development of floral organs [35,36,37,38]. When the three genes were mutated simultaneously, the floral organs were transformed into sepals. Additionally, the *B* and *C* genes need the *SEP* gene during pattern formation and organogenesis [19]. In determining the formation of lotus sepals, more experimental evidence is needed to confirm whether *NnAGL9* and *NnMADS6-like* have a similar function to that of the *NnSEP1* gene.

*AGL6* has a specific expression pattern in floral organs, but it varies slightly in different plant groups. Currently, the *AGL6* homologous gene is considered as one of the four core participants in the plant regulatory network for flower development (*SQUA*-like, *DEF/GLO*-like, *AG*-like and *AGL6*/*SEP1*-like) [24]. It mainly has the functions of regulating the flowering time, determining the characteristics of the flower meristem, determining the characteristics of flower organs and in the development of flower organs. Phylogenetic tree analysis showed that *AGL6* and *SEP* were sister branches [1,26]. The relationship between *AGL6* and *SEP* was relatively close [38]. In many species such as *Petunia × hybrida* and *Oryza sativa*, the function of *AGL6* was similar to the class *E* gene family [26,39]. However, it has a different function in *Oncidium flexuosum* and *Nymphaea tetragona*. The function of *AGL6* was similar to that of *AP1* that belongs to the class *A* gene family [40]. In this study, the pattern of the relatively high expression level of *NnMADS6-like* was similar to that of *NnSEP1* in the sepals and petals, while the expression patterns in the stamens and pistils were different. This hints that *NnMADS6-like* might have a function as a class *A* gene in *N. nucifera*.

The function of *AGL9* is also known as SEP3 in the model plant *Arabidopsis thaliana.* Phylogenetic analysis illustrated that *NnAGL9* has a close relationship with the *SEP* class. Smaczniak’s research showed that the binding between SEP3 and other MADS-box proteins can change the chromatin state [16,41,42,43]. It was also suggested that SEP3, as a pioneer transcription factor, modifies chromatin accessibility [44]. Based on a Y2H assay and gene expression patterns, a putative protein interaction showed that SEP3 interacted with AGL6 in the petals, stamens and pistils in *Arabidopsis* [45]. Our results also confirm that NnAGL9 interacts with NnMADS6-like in yeast, no matter who is the bait. It is suggested that NnAGL9 performs the function of SEP3 in the process of lotus organogenesis.

Although the lotus is a basal eudicot species in flowering plants, there are significant differences in the genes that control the sepals and petals. *NnMADS6-like* and *NnSEP1* might determine the formation of lotus sepals. To further understand the function of *NnSEP1*, *NnAGL9* and *NnMADS6-like*, we need to identify other factors and genes that are integrated in flowering development pathways and investigate how these regulators control floral organogenesis. Successfully deciphering the other class gene families and *SEP* genes will broaden our knowledge about the floral organ recognition control network in *N. nucifera.*

## Figures and Tables

**Table 1:** The primers used in this study.

Primer Name	Sequence (5′→3′)
AP	GGCCACGCGTCGACTAGTACTTTTTTTTTTTTTTTT
AUAP	GTACTAGTCGACGCGTGGCC
NnSEP1-full-F	ATGGGGAGAGGAAGGGTAGA
NnSEP1-RT-F	GGAAGCTGGATGAAAGTAGT
NnSEP1-RT-R	AAGCATCCACCCAGGAATAT
NnAGL9-RT-F	AGGGAGTCAGAGGAGCTGAG
NnAGL9-RT-R	CAATGCCCTGTTAGCTTCGC
NnMADS6-like-RT-F	AGCTAGGCAACGGAAGACAC
NnMADS6-like-RT-R	TCCCTCCGGTGGAACATAGT
NnActin-F	ACCACTGCTGAACGGGAAAT
NnActin-R	ATGGCTGGAATAGAACCTCA
1300-NnSEP1-GFP-F	GGATCCATGGGGAGAGGAAGGGTAGA
1300-NnSEP1-GFP-R	TCTAGAAAGCATCCACCCAGGAA
1300-Nn AGL9-GFP-F	GGATCCATGGGGAGAGGTAGGGTTGA
1300-Nn AGL9-GFP-R	TCTAGATGCCAGCCACCCAGGC
1300-NnMADS6-like-GFP-F	GGATCCATGGGAAGAGGACGAGTAGA
1300-NnMADS6-like-GFP-R	TCTAGAGAGAACCCATCCTTGAAT
AD-NnSEP1-F	CATATGGGGAGAGGAAGGGTAGAGCTGAA
AD-NnSEP1-R	GGATCCCAAGCATCCACCCAGGAATATAACCATT
AD-NnAGL9-F	CATATGGGGAGAGGAAGGGTAGAGCTGAA
AD-NnAGL9-R	GGATCCCTGCCAGCCACCCAGGCATATAACTA
AD-NnMADS6-like-F	CATATGGGGAGAGGAAGGGTAGAGCTG
AD-NnMADS6-like-R	GGATCCCGAGAACCCATCCTTGAATGAAGTTA

## Data Availability

This study did not report any other data.

## Supplementary Materials

The following are available online at https://www.mdpi.com/article/10.3390/plants10081629/s1, Figure S1: The wild Asian lotus was introduced from Weishan Lake in Shandong Province, China, Figure S2: The gene expression in different tissues, Figure S3: The flower buds of Asian lotus at four different growth stages. The lengths of buds from left to right are 2 cm, 3.8 cm, 6.5 cm and 9.5 cm; bar = 2 cm, Figure S4: The negative controls of protein interaction in vivo. Interactions were determined in yeast on selective medium SD/-Trp-Leu-His-Ade, and the experiments were repeated in triplicates with the same results.
