# Supplementary material for: Molecular Cloning and Expression Profile of Class E Genes Related to Sepal Development in Nelumbo nucifera
Source: Plants (Basel). 2021 Aug 9;10(8):1629. doi: 10.3390/plants10081629 (PMC8398900; doi:10.3390/plants10081629)
Supplement: Supplementary file 1 [file plants-10-01629-s001.zip › plants-1304504-supplementary.pdf]

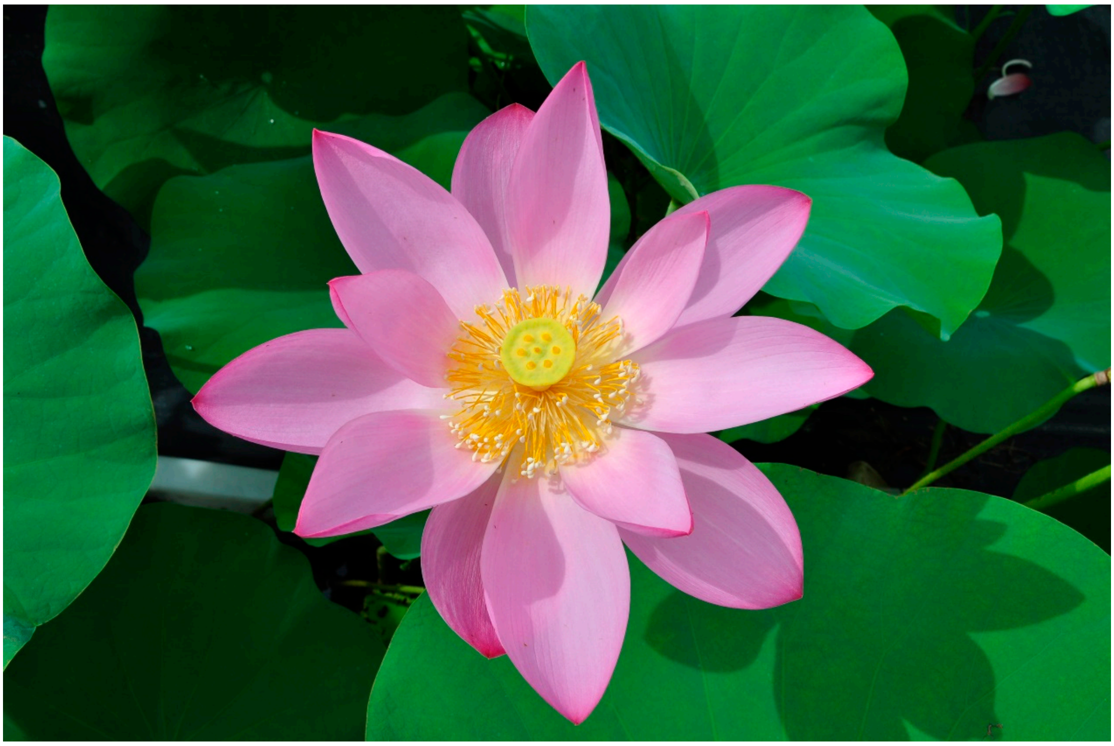

Figure S1. The wild Asian lotus was introduced from Weishan Lake of Shandong Province, China.

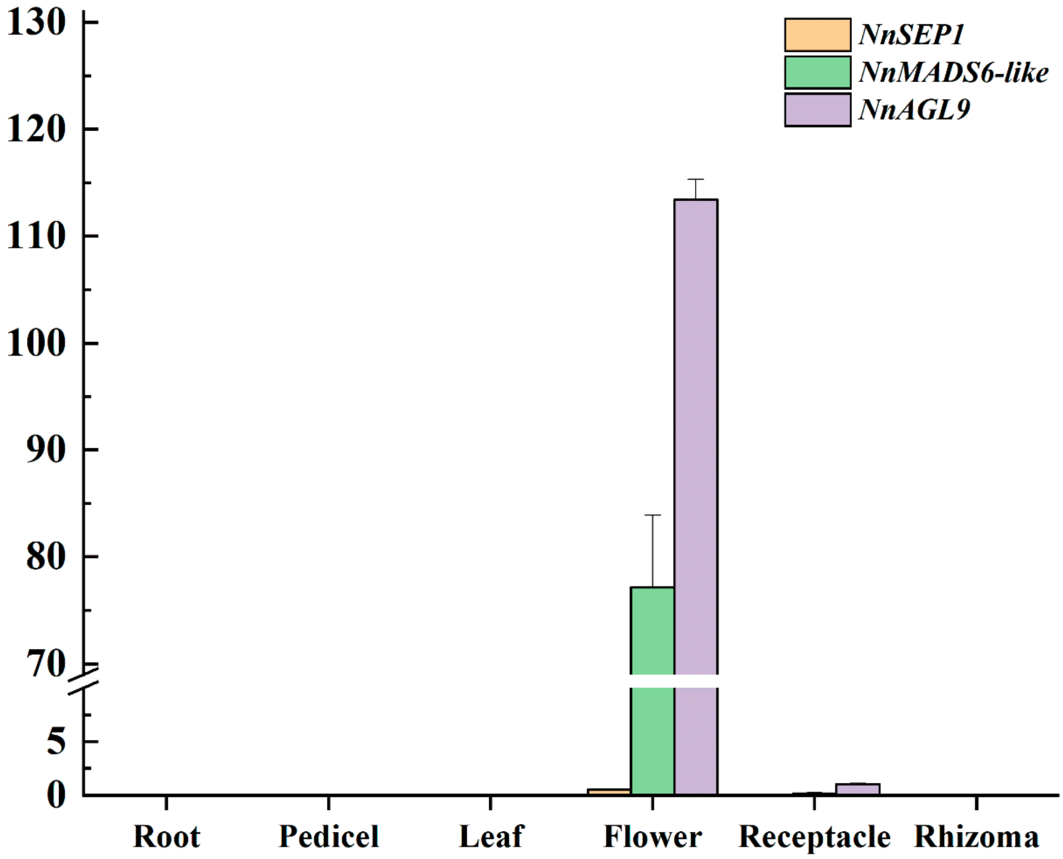

Figure S2. The gene expression in different tissues.

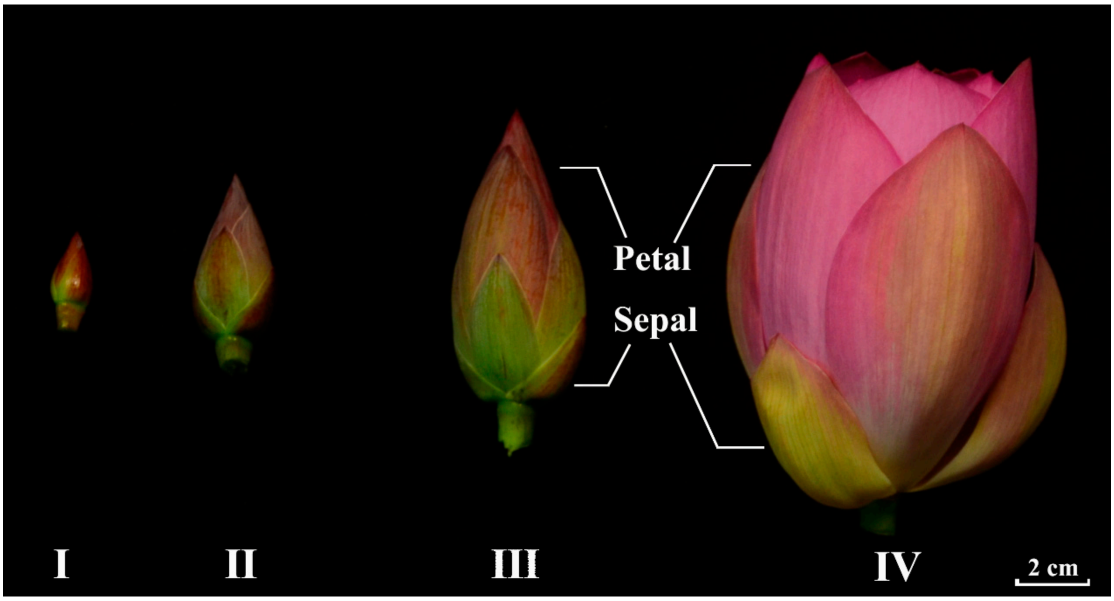

Figure S3. The flower buds of Asian lotus at four different growth stages. The lengths of buds from left to right are 2 cm, 3.8cm, 6.5cm and 9.5cm; Bar=2cm.

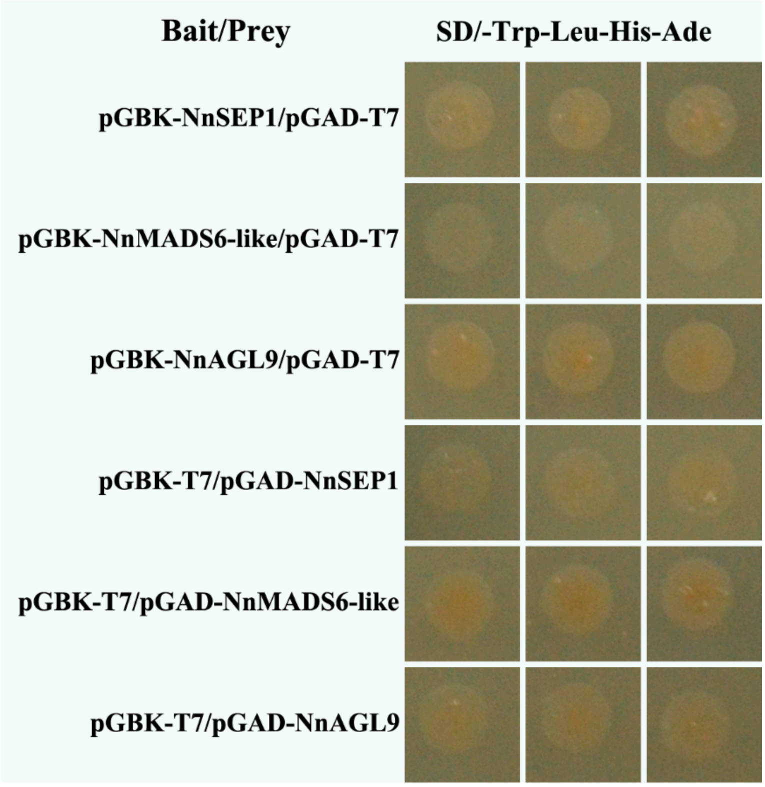

Figure S4. The negative controls of protein interaction in vivo. Interactions were determined in yeast on selective media SD/-Trp-Leu-His-Ade and the experiments were repeated in triplicates with the same results.
